# Association between gastric cancer and the risk of depression among South Korean adults

**DOI:** 10.1186/s12888-022-03847-w

**Published:** 2022-03-21

**Authors:** Sinyoung Kwon, Jinyeong Kim, Taeyeon Kim, Wonjeong Jeong, Eun-Cheol Park

**Affiliations:** 1 Premedical Cource, Yonsei University College of Medicine, Seoul, Republic of Korea; 2 Department of Preventive Medicine, Gachon University College of Medicine, 38-13, Dokjeom-ro 3beon-gil, Namdong-gu Incheon, 21565 Republic of Korea; 3 Institute of Health Services Research, Yonsei University, Seoul, Republic of Korea; 4 Department of Preventive Medicine, Yonsei University College of Medicine, Seoul, Republic of Korea

**Keywords:** Gastric cancer, Depression, Psychiatric conditions

## Abstract

**Objectives:**

The diagnosis and treatment of cancer are stressful events that could trigger psychological distress in a large number of cancer patients. The aim of this study was to examine the association between gastric cancer and the risk of new onset of depression among South Korean adults.

**Methods:**

Data from 12,664 participants aged over 40 years was derived from the National Health Service National Sample Cohort (2002–2013). The case cohort consists of patients who received a diagnosis of gastric cancer between 2002 and 2009, and the corresponding control group was selected through 1:1 propensity score matching (case: 6332, control: 6332). The new onset of depression was considered as the dependent variable. A Cox proportional hazards regression model was built to analyze the associations between variables in consideration.

**Results:**

Individuals with gastric cancer had a higher risk of new onset of depression than those without cancer (hazard ratio [HR] = 1.28, 95% confidence interval [CI] = 1.13–1.45.) Female gastric patients had a higher risk of depression compared to male patients (Female; HR = 1.89, 95% CI = 1.66–2.16, Male; HR = 1.25, 95% CI = 1.10–1.41). Gastric cancer patients in their 60s had the highest risk of new onset of depression compared to other age groups and no cancer group (HR = 1.61, 95% CI = 1.40–1.85). Gastric cancer patients who were previously diagnosed with depression prior to their diagnosis of cancer had a higher risk of new onset of depression than gastric cancer patients without antecedent diagnosis of depression (Past Depression (Yes); HR = 5.17, 95% CI = 4.10–6.51, Past Depression (No); HR = 1.35, CI = 1.21–1.51).

**Conclusions:**

The study identified a significant relationship between gastric cancer and depression among South Korean adults, suggesting that the diagnosis and treatment of gastric cancer increases the risk of new onset of depression, especially among female patients between 60 and 69 years old of high income and living in metropolitan regions. Pre-existing health conditions also appeared to be a risk factor. Thus, in consideration of treatment efficacy and patients’ quality of life, the results of the study emphasizes the need for attentive intervention, while distinguishing the most vulnerable groups.

## Background

Gastric cancer is a serious health problem deserving international attention, as it the fourth most common cancer and the second leading cause of cancer-related death in the world [1]. The issue is of utmost importance in South Korea, which had the highest prevalence of gastric cancer in the world [2]. In South Korea, gastric cancer had a crude incidence rate of 52.1 per 100,000 and crude mortality rate of 12.1 per 100,000 in 2020 [3]. Recently, as the diagnostic frequency of early gastric cancer has increased, the long-term outcomes and quality of life of the patients have become important issues [4]. Despite its importance, gastric cancer receives little attention from public health organizations, where only 0.2% of the budget was allocated to gastric cancer, and 10% of this amount was allocated to prevention research [5].

The diagnosis and treatment of cancer are stressful events that trigger psychological distress in large number of cancer patients [6]. Previous studies show that overall, 47.2 and 57% of patients with gastrointestinal cancer scored high on both anxiety and depression scales, respectively [6, 7]. Patients with gastric cancer experience psychological distress not only from the knowledge of their diagnosis, but from receiving surgery as well [6, 8]. Coexistence of depression and cancer in patients may engender other physical disease and hence have a detrimental effect on treatment efficacy and the recurrence rate of other various physical conditions [9].

Due to improved treatment methods and increased screening frequency, the survival of cancer patients has increased and the social support to care for their psychological well-being during or even after their treatment has become important [10]. Depression in cancer patients has been linked to decreased medical adherence, reduced quality of life, and even poor survival outcomes, indicating that psychosocial well-being is associated with survival benefits [11, 12]. Therefore, proper treatment of depression is necessary, and the goals of treatment should be avoiding adverse effects, improvement of symptoms, and ensuring long-term efficacy of treatment [13].

However, although proper short-term or long-term treatment for psychological distress is necessary, this is difficult to achieve as patients with depression exhibits varying patterns and severity of mood disturbances and clinical presentations [14]. Moreover, the diagnosis of depression can be challenging in cancer patients due to the overlapping of depressive symptoms with physical symptoms as a consequence of the illness and treatment [15]. Despite its pervasive impact on patients’ daily function and well-being, psychical problems are usually undertreated and overlooked among cancer patients [15].

Therefore, it is necessary to investigate the association between gastric cancer and the risk of depression to demonstrate the importance of alleviating the burden of depression. We hypothesized that individuals with gastric cancer are at a higher risk of depression than those who do not have cancer. Moreover, we also performed subgroup analysis in order to identify the most vulnerable populations, with regards to how this may inform health policy makers.

## Methods

### Data and study participants

The data for this study were obtained from the 2002–2013 National Health Insurance Service National Sample Cohort (NHIS-NSC), by random sampling. The NHIS-NSC data includes medical claims from 1,025,340 individuals, accounting for 2% of the South Korean population. The data provides information on various socioeconomic variables such as individuals’ gender, residential area, type of health insurance, income level, etc. Medical records of individuals provides information about participants’ medical bill claimed by medical service providers – more specifically, the participant’s electronic medical treatment bills, bill details, details of disease and details of prescriptions.

**Fig. 1:**
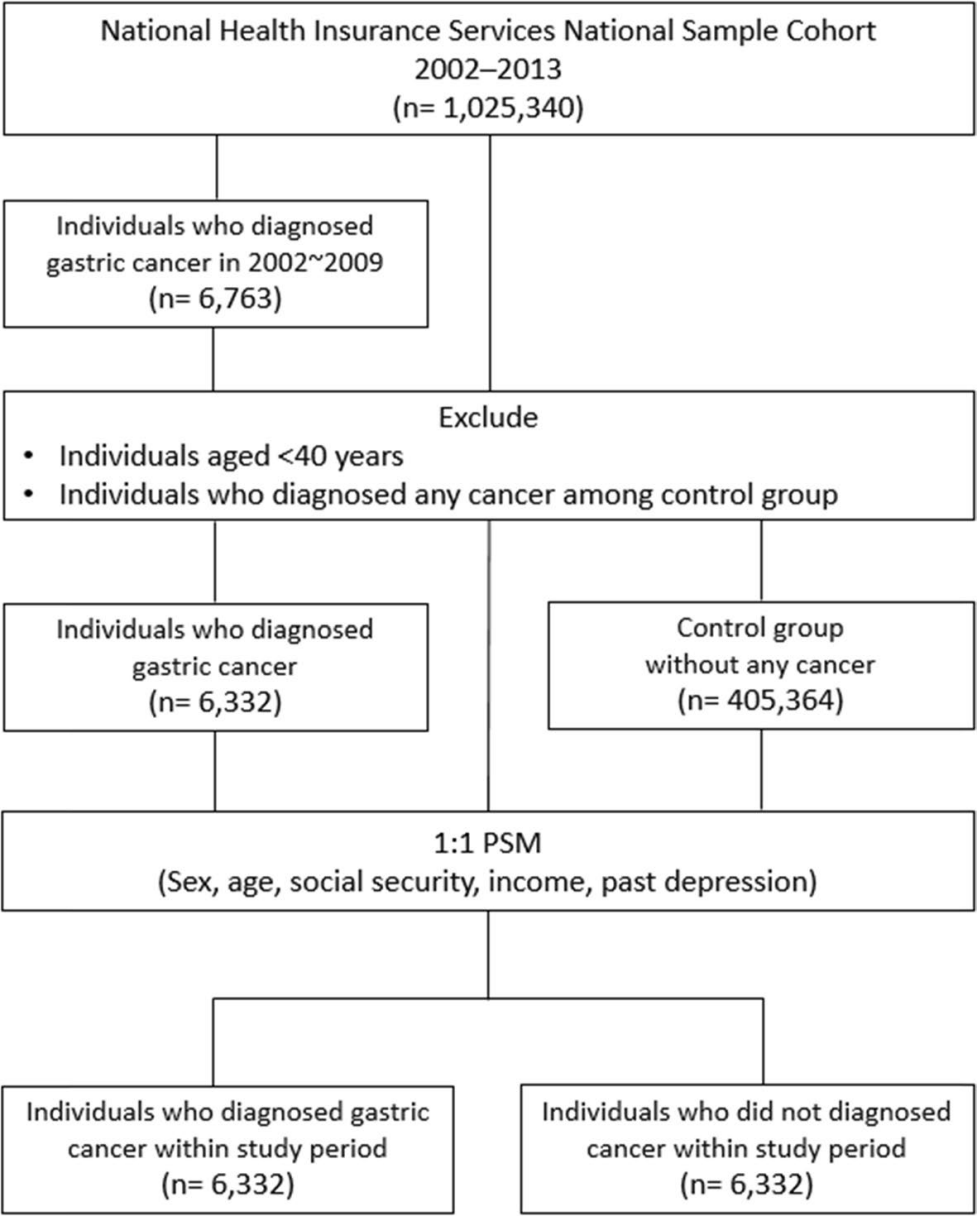
Flowchart of the participant selection

Individuals over the age of 40 years were selected for the study [16]. Patients diagnosed with gastric cancer from 2002 to 2009 were included in the case cohort, and the corresponding control group was derived from the general population who weren’t diagnosed with any type of cancer within the given time interval, and thereby individuals diagnosed with cancers other than gastric cancer were excluded. The year the individual was diagnosed with gastric cancer (from 2002 to 2009) was considered as the year of cohort entry for the case cohort group, while for the control group, the year of cohort entry was considered as the year in which most number of claims were made between 2002 and 2009. We then performed 1:1 propensity score matching (matching variables: age, sex, social security, income, and past depression) to generate a matched control group to the case cohort (Fig. 1). Consequently, a total of 12,664 individuals were included in the final study (case: 6332, control: 6332). All the data is available in the database of the Korean National Health Insurance Sharing Service (https://nhiss.nhis.or.kr) and can be accessed upon reasonable request. This study adhered to the tenets of the Declaration of Helsinki and was based on routinely collected administrative and claims data. This study was reviewed and approved by the International Review Board of Yonsei University’s Health System (IRB number: 4–2021-0705).

### Variables

The new onset of depression (International Classification of Diseases, 10th revision [ICD-10] code: F32, F33) according to medical claims data is the dependent variable. Major Depressive Disorder (MDD) that was clinically diagnosed after their year of cohort entry (the year of gastric cancer diagnosis for the case cohort) was considered as the ‘new’ onset depression. If the patient had been diagnosed with depression prior to the date of cohort entry, this was taken into consideration as a variable of interest (‘past depression’). Throughout the paper, the dependent variable is referred to and is considered as the ‘new’ onset of depression, for it can also imply a ‘relapse’ or ‘recurrence’. Overall, the diagnosis of depression was analyzed within the follow-up period or by the end of the study period (12/31/2013).

The primary independent variable is the diagnosis of gastric cancer that the patient had received (ICD-10: C16). Other variables of interest – or covariates - included sex, social security, income, region, disability, diabetes (ICD-10: E10, E11, E12, E13, E14), hypertension (ICD-10: I10, I11, I12, I13, I14, I15), and past depression. In South Korea, diabetes and hypertension prevalence is increasing, and are considered as **a** chronic condition that is a contributing risk factor to other diseases [17, 18]. As aforementioned, past depression refers to those who had already been diagnosed with depression prior to their year of cohort entry (the year of gastric cancer diagnosis for the gastric cancer patients).

### Statistical analysis

The chi-square test was carried out to investigate the general characteristics of the study population. Propensity score matching (1:1) was used to match subjects with and without gastric cancer. A Cox proportional hazards regression model was used to calculate the association between gastric cancer and the risk of depression. Subgroup analysis was conducted to investigate this association between the new onset of depression and covariates according to gastric cancer. We stratified the participants into subsets based on covariates to examine how the relationship between gastric cancer and depression is affected by other relevant environmental or biological conditions. The subgroup analysis could be useful in determining which characteristics of the individuals serve as the effect modifier in explaining the relationship between gastric cancer and depression. The differences with a *p*-value < 0.05 were considered statistically significant. All data analyses were carried out using SAS 9.4 software (SAS Institute Inc., Cary, NC, USA).

## Results

Table 1 presents the general characteristics of the study population. Among the 12,664 participants, as a propensity matching of 1:1 was performed, the case cohort as well as the matched control each consisted of 6332 (50.0%) individuals. In both cohorts, 4147 (65.5%) individuals were male, and 225 (3.6%) individuals had been previously diagnosed with depression, meaning that 225 gastric cancer patients had been diagnosed with depression prior to their diagnosis of cancer.

**Table 1:** General characteristics of the study population ^a^

Variables		Total		Gastric Cancer				
				Yes		No		*P*-value
Total		12,664	(100.0)	6332	(50.0)	6332	(50.0)	
**Sex**							1.0000	
	Male	8294	(65.5)	4147	(65.5)	4147	(65.5)	
	Female	4370	(34.5)	2185	(34.5)	2185	(34.5)	
**Age**							1.0000	
	40–49	1934	(15.3)	967	(15.3)	967	(15.3)	
	50–59	2892	(22.8)	1446	(22.8)	1446	(22.8)	
	60–69	4250	(33.6)	2125	(33.6)	2125	(33.6)	
	≥70	3588	(28.3)	1794	(28.3)	1794	(28.3)	
**Social Security**							1.0000	
	Health Insurance (Corporate)	5118	(40.4)	2559	(40.4)	2559	(40.4)	
	Health Insurance (Regional)	7172	(56.6)	3586	(56.6)	3586	(56.6)	
	Medical aid	374	(3.0)	187	(3.0)	187	(3.0)	
**Income**							1.0000	
	Low	2048	(16.2)	1024	(16.2)	1024	(16.2)	
	Middle	4890	(38.6)	2445	(38.6)	2445	(38.6)	
	High	5726	(45.2)	2863	(45.2)	2863	(45.2)	
**Region**							< 0.0001	
	Metropolitan	4959	(39.2)	2345	(37.0)	2614	(41.3)	
	City	3223	(25.5)	1589	(25.1)	1634	(25.8)	
	Rural	4482	(35.4)	2398	(37.9)	2084	(32.9)	
**Disability**							0.0003	
	Yes	1273	(10.1)	575	(9.1)	698	(11.0)	
	No	11,391	(89.9)	5757	(90.9)	5634	(89.0)	
**Diabetes**							0.0002	
	Yes	1572	(12.4)	718	(11.3)	854	(13.5)	
	No	11,092	(87.6)	5614	(88.7)	5478	(86.5)	
**Hypertension**							< 0.0001	
	Yes	2924	(23.1)	1178	(18.6)	1746	(27.6)	
	No	9740	(76.9)	5154	(81.4)	4586	(72.4)	
**Past Depression**							1.0000	
	Yes	450	(3.6)	225	(3.6)	225	(3.6)	
	No	12,214	(96.4)	6107	(96.4)	6107	(96.4)	
**Year of Cohort entry**							< 0.0001	
	2002	1718	(13.6)	1417	(22.4)	301	(4.8)	
	2003	1129	(8.9)	823	(13.0)	306	(4.8)	
	2004	1032	(8.1)	730	(11.5)	302	(4.8)	
	2005	1109	(8.8)	718	(11.3)	391	(6.2)	
	2006	1066	(8.4)	648	(10.2)	418	(6.6)	
	2007	1524	(12.0)	622	(9.8)	902	(14.2)	
	2008	2283	(18.0)	681	(10.8)	1602	(25.3)	
	2009	2803	(22.1)	693	(10.9)	2110	(33.3)	

^a^The matching variables were sex, age, social security, income, and past depression.

Table 2 shows the association between gastric cancer and the risk of new onset of depression after controlling for all covariates. Individuals with gastric cancer had higher risk of new onset of depression than those with no cancer (HR = 1.28, 95% confidence interval [CI] = 1.13–1.45), thus confirming our hypothesis. Female patients had a higher risk of new onset of depression than male patients (HR = 1.55, 95% CI = 1.39–1.73). Individuals with disabilities had higher risk of new onset of depression than those without disability (HR = 1.29, 95% CI = 1.09–1.53). Individuals previously dealing with diabetes had a higher risk of new onset of depression than those who weren’t (HR = 1.21, 95% CI = 1.03–1.42). Individuals diagnosed with hypertension had higher risk of new onset of depression than those who weren’t (HR = 1.22, 95% CI = 1.07–1.39).

**Table 2:** The association between participant characteristics, including gastric cancer, and depression

Variables		Risk of Depression				
		HR	95% CI			P-value
**Gastric Cancer**					< 0.0001	
	Yes	1.28	(1.13	–	1.45)	
	No	1.00				
**Sex**					< 0.0001	
	Male	1.00				
	Female	1.55	(1.39	–	1.73)	
**Age**					0.0455	
	40–49	1.00				
	50–59	0.90	(0.75	–	1.08)	
	60–69	1.11	(0.93	–	1.31)	
	≥70	0.98	(0.82	–	1.18)	
**Social Security**					0.2477	
	Health Insurance (Corporate)	1.00				
	Health Insurance (Regional)	1.00	(0.89	–	1.11)	
	Medical aid	0.71	(0.47	–	1.07)	
**Income**					0.2487	
	Low	1.00				
	Middle	0.90	(0.76	–	1.06)	
	High	0.98	(0.83	–	1.16)	
**Region**					0.1075	
	Metropolitan	1.00				
	City	0.90	(0.78	–	1.03)	
	Rural	0.88	(0.78	–	1.00)	
**Disability**					0.0035	
	Yes	1.29	(1.09	–	1.53)	
	No	1.00				
**Diabetes**					0.0241	
	Yes	1.21	(1.03	–	1.42)	
	No	1.00				
**Hypertension**					0.003	
	Yes	1.22	(1.07	–	1.39)	
	No	1.00				
**Past Depression**					< 0.0001	
	Yes	5.34	(4.52	–	6.30)	
	No	1.00				
**Year of Cohort entry**					< 0.0001	
	2002	1.89	(1.55	–	2.29)	
	2003	1.39	(1.11	–	1.73)	
	2004	1.38	(1.11	–	1.73)	
	2005	1.12	(0.89	–	1.41)	
	2006	1.10	(0.87	–	1.38)	
	2007	1.14	(0.93	–	1.39)	
	2008	1.06	(0.88	–	1.28)	
	2009	1.00				

Those who had been given a diagnosis of depression prior to date of cohort entry had a higher risk of a new onset than those who hadn’t. (HR = 5.34, 95% CI = 4.52–6.30). The risk of new onset of depression also appeared significantly different according to the year of cohort entry, with the risk being notably high for the first few years. However, this appears so due to the fact that individuals with an early year of cohort entry were followed for a greater period of time.

Table 3 shows the results of subgroup analysis, where the case cohort and the controls were further categorized into subgroups by relevant covariates. Hence, we were able to ascertain that the effect the diagnosis of gastric cancer has on the new onset of depression is maintained - as previously shown in Table 2 – while examining for any confounding variables.

**Table 3:** Subgroup analysis of the association between gastric cancer and risk of depression

	Gastric Cancer				
	Yes			No	
	Adjusted HR	95% CI		Adjusted HR	P-value
		(Lower-Upper)			
**Sex**					
Male	1.31	(1.12 – 1.55)		1.00	0.001
Female	1.25	(1.04 – 1.50)		1.00	0.0192
**Age**					
40–49	1.44	(1.03 – 2.01)		1.00	0.0312
50–59	1.68	(1.25 – 2.26)		1.00	0.0006
60–69	1.20	(0.97 – 1.47)		1.00	0.088
≥70	1.07	(0.86 – 1.32)		1.00	0.5465
**Social Security**					
Health Insurance (Corporate)	1.55	(1.28 – 1.88)		1.00	< 0.0001
Health Insurance (Regional)	1.14	(0.97 – 1.34)		1.00	0.1227
Medical aid	0.54	(0.24 – 1.22)		1.00	0.1379
**Income**					
Low	1.14	(0.85 – 1.53)		1.00	0.3708
Middle	1.47	(1.20 – 1.80)		1.00	0.0002
High	1.19	(0.99 – 1.42)		1.00	0.0595
**Region**					
Metropolitan	1.25	(1.04 – 1.50)		1.00	0.0194
City	1.60	(1.24 – 2.05)		1.00	0.0003
Rural	1.12	(0.91 – 1.38)		1.00	0.2959
**Disability**					
Yes	1.06	(0.75 – 1.49)		1.00	0.7507
No	1.31	(1.15 – 1.50)		1.00	< 0.0001
**Diabetes**					
Yes	1.34	(0.98 – 1.83)		1.00	0.0678
No	1.27	(1.11 – 1.45)		1.00	0.0004
**Hypertension**					
Yes	1.04	(0.83 – 1.32)		1.00	0.7254
No	1.36	(1.18 – 1.57)		1.00	< 0.0001
**Past Depression**					
Yes	0.72	(0.51 – 1.00)		1.00	0.0471
No	1.41	(1.24 – 1.61)		1.00	< 0.0001

Noteworthy results are that of cohorts categorized by ‘social security (medical aid)’ and ‘past depression (yes)’. Gastric cancer patients whose social security is covered by medical aid had a lower risk of new onset of depression than individuals without gastric cancer whose social security is covered by medical aid (HR = 0.54, 95% CI = 0.24–1.22). Although the results aren’t statistically significant, it does provide evidence to about how the cost burden of diagnosis and treatment can be a significant contributor to the mental stability of gastric cancer patients.

Among individuals who had never been diagnosed with depression, gastric cancer patients had significantly higher risk of new onset depression than those with no cancer (HR = 1.41, 95% CI = 1.24–1.61). Meanwhile, gastric cancer patients who were previously diagnosed with depression prior to their diagnosis of cancer had a lower risk of new onset of depression than individuals without gastric cancer. (HR = 0.72, 95% CI = 0.51–1.00). Such results have interesting implications which are further explained under ‘discussions’.

Table 4 shows the comparison of risk of new onset depression between subgroups of gastric cancer patients and the control group (‘no cancer’). Its purpose is to identify characteristics of vulnerable subgroups within the case cohort. While comparison with the reference group – the ‘no cancer group’ – is present in the analysis, the crux of Table 4 is the comparison between the subgroups of the case cohorts.

**Table 4:** Analysis of risk of new onset depression between subgroups of gastric cancer patients and no cancer group

Variables			Risk of Depression		
			HR^a^	95% CI	p-value
**Sex**					< 0.0001
	Gastric Cancer	Male	1.25	(1.10 – 1.41)	
		Female	1.89	(1.66 – 2.16)	
	No Cancer		1.00		
**Age**					< 0.0001
	Gastric Cancer	40–49	1.45	(1.20 – 1.77)	
		50–59	1.42	(1.20 – 1.68)	
		60–69	1.61	(1.40 – 1.85)	
		≥ 70	1.34	(1.14 – 1.57)	
	No Cancer		1.00		
**Social Security**					< 0.0001
	Gastric Cancer	Health Insurance (Corporate)	1.55	(1.36 – 1.78)	
		Health Insurance (Regional)	1.45	(1.28 – 1.64)	
		Medical aid	0.60	(0.32 – 1.11)	
	No Cancer		1.00		
**Income**					< 0.0001
	Gastric Cancer	Low	1.32	(1.08– 1.61)	
		Middle	1.38	(1.20 – 1.59)	
		High	1.59	(1.40 – 1.81)	
	No Cancer		1.00		
**Region**					< 0.0001
	Gastric Cancer	Metropolitan	1.59	(1.38 – 1.82)	
		City	1.47	(1.25 – 1.72)	
		Rural	1.35	(1.17 – 1.55)	
	No Cancer		1.00		
**Disability**					< 0.0001
	Gastric Cancer	Yes	1.58	(1.25 – 2.00)	
		No	1.45	(1.30 – 1.62)	
	No Cancer		1.00		
**Diabetes**					< 0.0001
	Gastric Cancer	Yes	1.67	(1.35 – 2.06)	
		No	1.44	(1.29 – 1.61)	
	No Cancer		1.00		
**Hypertension**					< 0.0001
	Gastric Cancer	Yes	1.55	(1.30 – 1.85)	
		No	1.45	(1.29 – 1.62)	
	No Cancer		1.00		
**Past Depression**					< 0.0001
	Gastric Cancer	Yes	5.17	(4.10 – 6.51)	
		No	1.35	(1.21 – 1.51)	
	No Cancer		1.00		

^a^ Crude Hazard ratio

Female gastric cancer patients had higher risk of new onset depression than male gastric cancer patients (Female; HR = 1.89, 95% CI = 1.66–2.16, Male; HR = 1.25, 95% CI = 1.10–1.41). Gastric cancer patients in their 60s had the highest risk of new onset depression compared to other age groups and the control population (HR = 1.61, 95% CI = 1.40–1.85). Gastric cancer patients older than 70 years old showed the lowest risk of new onset of depression among other age groups (HR = 1.34, 95% CI = 1.14–1.57).

Gastric cancer patients whose social security is covered by medical aid showed lower risk of new onset depression than those with corporate or regional health insurance. The results are also consistent with that of Table 3, for patients whose social security was covered by medical aid had a lower risk of new onset of depression compared to the control. (Medical aid; HR = 0.60, 95% CI = 0.32–1.11, Health Insurance (Corporate); HR = 1.55, 95% CI = 1.36–1.78), Health Insurance (Regional); HR = 1.45, 95% CI = 1.28–1.64). Gastric cancer patients with high income showed higher risk of new onset depression than those with low or middle level of income (High Income; HR = 1.59, 95% CI = 1.40–1.81, Low Income; HR = 1.32, 95% CI = 1.08–1.61), Middle Income; HR = 1.38, 95% CI = 1.20–1.59)). Gastric cancer patients living in metropolitan had the highest risk of new onset depression among patients living in metropolitan, city, rural areas (Metropolitan; HR = 1.59, 95% CI = 1.38–1.82, City; HR = 1.47, 95% CI = 1.25–1.72, Rural; HR = 1.35, 95% CI = 1.17–1.55).

Gastric cancer patients with disability had a higher risk of new onset of depression than those without disability (Disability (Yes); HR = 1.58, 95% CI = 1.25–2.00, Disability (No); HR = 1.45, 95% CI = 1.30–1.62). Gastric cancer patients previously diagnosed with diabetes had a higher risk of new onset of depression than those with no diabetes (Diabetes (Yes); HR = 1.67, 95% CI = 1.35–2.06, Diabetes (No); HR = 1.44, 95% CI = 1.29–1.61). Gastric cancer patients previously diagnosed with hypertension showed higher risk of new onset of depression than those with no hypertension (Hypertension (Yes); HR = 1.55, 95% CI = 1.30–1.85, Hypertension (No); HR = 1.35, 95% CI = 1.29–1.62). Gastric cancer patients who were previously diagnosed with depression prior to their diagnosis of cancer had a higher risk of new onset of depression than gastric cancer patients without depression diagnosis history (Past Depression (Yes); HR = 5.17, 95% CI = 4.10–6.51, Past Depression (No); HR = 1.35, CI = 1.21–1.51).

## Discussion

This study examined the association between gastric cancer and depression risk among South Korean adults, performing analysis at different angles to obtain a profound understanding.

Based on our results, individuals with gastric cancer had higher risk of depression than those without cancer. The pattern was shown to be fairly consistent when subgroup analysis was performed, excluding the factor of ‘past depression’ as shown in Table 3. Gastric cancer patients who were previously diagnosed with depression had a lower new onset of depression after their diagnosis of cancer compared to the control group. However, in Table 4, the results seem to exhibit a contradictory pattern, where gastric cancer patients who were previously diagnosed with depression were at a much greater risk of being diagnosed again compared to gastric cancer patients who weren’t diagnosed with depression prior to their diagnosis of cancer. This discrepancy seems to be due to the method of analysis was performed. In Table 3, each case cohort sorted by whether they were previously diagnosed with depression were compared to controls who were correspondingly diagnosed or not diagnosed with depression prior to their date of cohort entry. In Table 4, the reference group was the entire control population. Rather, the discrepancy informs us that amongst patients suffering from depression, cancer diagnosis does not have a significant effect on their recurrence or relapse. Instead, the results indicate that the opposite phenomenon is observed. Other studies also seem to suggest that cancer patients who previously received mental health treatment are at a lower risk of developing depression [19]. On the other hand, amongst gastric cancer patients, prior diagnosis of depression seems to predict a higher risk of new onset of depression. This pattern fits the “stress-diathesis model”. Stress-diathesis theory of depression states that the effect of stress- in this case the diagnosis of gastric cancer- on the depression risk are dependent on the diathesis or vulnerability that can be explained as genetic, psychological, biological factors. This suggests that the traumatic experience of gastric cancer diagnosis might not have had the same effect on those who had been or not been through depression. Hence, if a gastric cancer patient had previously suffered from depression, they should be categorized as ‘vulnerable’ for relapse or recurrence throughout their treatment process and clinical service might as well emphasize prevention research and programs for prevention of depression.

Nevertheless, the results still suggest that past history of depression is one of the risk factors of depression that should be considered and treated; however, it is hard to determine depression for cancer patients with severe forms of cancer [15]. Moreover, as depression is highly associated with poor adherence to cancer treatment and poor cancer survival, providing proper care in time is important [20]. However, still among those without past depression, those who had gastric cancer had a higher risk of depression than those without cancer. In South Korea, the access rate to health care service for depression has been reported to be low, which shows that annual treatment rate for major depressive was only 56.3% [21]. Therefore, there might be people who did not diagnosed past depression although they had depressive symptoms. The importance of treating those who have past depression must be emphasized.

Our results also identify gastric cancer patients with comorbidity – namely diabetes and hypertension – to be at a higher risk of new onset of depression. This is consistent with the results of numerous other studies whose results demonstrate that pre-existing health conditions heighten one’s risk of depression when diagnosed with cancer [19, 22]. Our results also identify female gastric cancer patients between 60 and 69 years old, of high income and living in metropolitan areas – in contrast to rural regions – to be most vulnerable to new onset of depression when diagnosed with gastric cancer. The mentioned subgroup may experience greater stress due to barriers to return to work [3, 23]. As cancer patients lose their jobs due to cancer treatment or encounter heavy expenditure, the concerns grow over time [23, 24].

Meanwhile, although it is known that rural residents are characterized as a vulnerable population, the results show that they are more immune to the new onset of depression when diagnosed with gastric cancer than populations living in metropolitan areas. However, the discrepancy is a dubious one, for in Korea, individuals living in rural areas tend to receive cancer treatment in hospitals of metropolitan areas. On the other hand, for other less urgent conditions, they receive treatment in hospitals nearby their residential area. Hence, travelling from rural areas to metropolitan cities may already place a heavy burden. Moreover, due to socioeconomic differences between such regions, access to diagnosis and treatment of depression may be lacking, and hence decrease the validity of the data collected [25, 26].. Hence this implies that there might be some underestimated results. Moreover, among cancer patients and cancer survivors, depressive symptoms are frequently underdiagnosed or underestimated, which might result in inadequate treatment [14]. Moreover, untreated depression might be associated with exacerbation of symptoms burden, lack of exercise, worsened quality of life, and even increased psychological burden on the family members [14, 27]. This could lead to increased healthcare utilization and expenditure that could increase the burden on healthcare and lead to a vicious cycle of depression [14, 28].

It should be noted that the current study has several limitations. First, as this study used a retrospective cohort and claim data collected to provide payment for both patient and providers based on medical utilization, factors related to unhealthy behaviors such as smoking status or frequency of alcohol consumption that could affect the physical conditions, were not examined. Second, the risk of depression might have been underreported as there might be some patients who could not undergo investigations to be diagnosed with depression because of the severity of their cancer [15]. Moreover, we could not include those who suffered depressive symptoms but were not diagnosed with depression because while depression is common in cancer patients, it is not commonly diagnosed as it is considered relatively less important than cancer treatment [14]. Third, we could not adjust the severity of gastric cancer due to lack of data. As this study used claim data, the stage of cancer was not known. Lastly, other psychological disorders or other cancers were not included in this study. Further research on various psychological disorders is needed.

The strengths of our study are as follows. This study used national sampling cohort data. As these data could represent the entire population of South Korea, we could provide the evidence on the importance of managing patients with gastric cancer, protecting them from depression, and providing them timely care. Moreover, as the data covered nearly 10 years, we could show the long-term association between these variables. In addition, this study analyzed the association between the time span from the first diagnosis of gastric cancer and the risk of depression, which could provide accurate information about the conditions of patients. Lastly, this study highlights the need for diagnosis and treatment of depression, which may often be underestimated, among cancer patients.

## Conclusions

The current study identified a significant relationship between gastric cancer and the risk of depression among South Korean adults. The risk of depression was higher among those who were diagnosed with gastric cancer, compared to those without cancer. Our results identify female gastric patients between 60 and 69 years old of high income living in metropolitan areas to be most vulnerable to new onset of depression when diagnosed with gastric cancer. However, more research and consideration should be given as to whether the results reflect reality or are a product of better documentation and more advanced health services. Comorbidity – including previous diagnosis of depression – increased one’s risk of a new onset of depression after diagnosis of gastric cancer. As preventing depression could improve the public’s health among persons with chronic medical conditions, prevention of mental health is necessary [29]. This implies that caution should be taken to prevent depression in those who are recently diagnosed with gastric cancer. Our study suggests that interventions should be designed to alleviate the risk of depression, provide proper treatment for depression, and highlight the importance of managing depression in cancer patients.

## Data Availability

All data are available from the database of the Korean National Health Insurance Sharing Service (https://nhiss.nhis.or.kr) and can be accessed upon reasonable request.

## Abbreviations

ICD-10: International Classification of Diseases, 10th revision
NHIS-NSC: National Health Insurance Service National Sample Cohort
